# After all, it is an outdoor sport: Meta-analytic evidence for negative associations between wind compensation points and round scores in ski jumping competitions

**DOI:** 10.1371/journal.pone.0238101

**Published:** 2020-08-24

**Authors:** Jakob Pietschnig, Marie Pellegrini, Junia Sophia Nur Eder, Magdalena Siegel

**Affiliations:** Department of Developmental and Educational Psychology, Faculty of Psychology, University of Vienna, Vienna, Austria; University of Zurich, SWITZERLAND

## Abstract

Wind conditions are well-known to affect results of ski jumping competitions. To alleviate effects of different head or tail winds and differences in inrun length due to jury or coaches’ decisions, the FIS (Féderation Internationale de Ski) has adopted a wind and gate compensation system since January 2010. However, the accuracy and fairness of the resulting compensation points are often questioned by athletes, spectators, and media commentators alike but have not yet been thoroughly investigated. In the present meta-analysis, we present evidence for systematic negative associations of wind points but positive associations of gate points with round scores across all World Cup and World Championship competitions of men in the ski jumping seasons 2016/2017 and 2017/2018 (*k* = 35 and 30, respectively). Moreover, our results indicate that absolute correlations between wind points and round scores increased in presence of larger wind point variability, thus indicating lower accuracy of compensation systems when conditions are more variable. Additionally, there was a trend for larger wind point malcompensations on larger jumping hills. Our results suggest that the current wind point compensation formula as well as gate factors should be reevaluated to prevent systematically biased point awards within and across competitions.

## Introduction

Differing weather and wind conditions are well-known to exert meaningful and potentially competition-distorting influences on the results of ski jumping [1]. Typically, it is assumed, that head winds lead to longer jumps and consequently higher points, whilst tail winds lead to shorter jumps and consequently lower points. Importantly, recent results of computer simulation models have shown, that this assumption might not be entirely justified, because head winds may be disadvantageous in the initial phase of the jump, but beneficial in the later phase, whilst for tail wind it is the other way around [2]. In general, it has been argued that although weather conditions influence individual competition results, results of entire World Cup seasons should be comparatively little affected because differing conditions should even each other out over the course of many competitions. Still, results of isolated important events such as the Olympics, the World Championship, or the Four Hills Tournament rely on the results of only two rounds of jumps within one or only few competitions and may be therefore substantially influenced by weather conditions.

To account and correct for wind effects, the FIS (Féderation Internationale de Ski) introduced a compensation system which has been adopted in January 2010 [3] and revised in 2016 (see [4] and the Appendix of [2]). By awarding (or deducting) points, according to the formula

Δw=TWS*(HS‐36)/20

where, Δ*w* = wind effect on jumping distance in meters, TWS = tangential wind speed in meters per second, and HS = hill size in meters, the effects of (un-)favorable conditions should be compensated for [3, 4]. Importantly, TWS is calculated as a weighted average of wind speeds at different measurement points on the hill, to account for differential influences of wind in different phases of the jump. Depending on the hill size, the wind is typically measured on 5 (normal hills), 7 (large hills) or 10 points (ski-flying hills).Within the same initiative, the FIS introduced gate compensation points for within-competition variations of inrun length to further increase comparability and fairness of ski jumping results. Thereby, athletes are awarded (or deducted) a fixed number of points on a given hill if they start from a lower (or higher) gate than the jury-defined starting gate. This approach has been deemed useful to avoid (i) round repetitions due to mid-round gate changes because of jury decisions, (ii) dangerously long jumps of high-performing athletes, and (iii) short jumps of athletes who are not expected to perform on a comparable level as the top athletes.

Although these compensation systems have initially been met with some skepticism on behalf of the athletes [3], sport associations, and the news media [5], the wind and gate compensation system has by now become the unanimous standard of ski jumping competitions. However, the fairness and adequacy of these systems continue to be called into question [6]. In fact, computer simulations [7] and empirical analyses of ski jumper data in three seasons up to 2013/2014 [8] suggest that the wind and gate point systems may yield inappropriate compensation. It has been reported, that the wind compensation system seemed to malcompensate round scores substantially, whilst the gate point system showed evidence for slight overcompensation of scores [8]. Moreover, it has been shown that effects of head and tailwind are not linear over the entire distance of a jump [7]. This has led, among others, to the improvement of wind compensation points by introducing weighting procedures to account for the non-linearity [4]. Furthermore, recent computer models [2] suggest that head and tailwinds may have differential effects on jumping performance, depending on their encounter on the beginning or the end of a jump. It seems likely that amends to the wind and gate compensation system in 2016 [4] may have improved the suboptimal properties of the system that has been in place before. However, conclusive empirical data that corroborate this interpretation is to date unavailable.

Here, we present a first meta-analytical account of all World Cup and World Championship competitions of men in the ski jumping seasons 2016/2017 and 2017/2018. By means of an empirical meta-analytical approach, we plan to (i) assess accuracy and fairness of wind and gate compensation points by synthesizing bivariate within-competition associations of wind or gate points with round scores (i.e., if the compensation systems are fair, there should be no meaningful systematic association between these variables), (ii) investigate the variability of effect sizes between competitions, thus providing evidence for potential non-uniformity of malcompensation across different events, (iii) examine effects of hill size and scoring mode (i.e., standard single vs. team or KO scoring) on associations between wind or gate points and round scores, and (iv) assess influences of wind point variability on the strength of associations between wind points and round scores.

## Methods

### Data collection

We obtained all World Cup and World Championships results of men in the 2016/2017 and 2017/2018 ski jumping seasons (i.e., covering events from November 25, 2016 to March 26, 2017 and November 18, 2017 to March 25, 2018) from the FIS database (https://data.fis-ski.com/ski-jumping/results.html). A PRISMA flow-chart of our data collection and the PRISMA checklist can be found in the online supplementary S1 Fig and S1 Checklist. We included only results from competitive jumps (i.e., no training and qualification jumps were eligible) and categorized events according to competition modes (i.e., individual jumps, team jumps, individual flying, team flying, KO competition). For the individual competitions, we calculated (i) zero-order Pearson correlations of wind points and (ii) non-parametric Spearman correlation of gate points with individual total scores for each round separately and for both rounds, thus obtaining six correlations for each event (i.e., first vs. second vs. both rounds by wind vs. gate points). For both round calculations, only data of athletes that qualified for participation in the second round were included.

### Data analysis

We used a meta-analytical approach to synthesize associations of wind and gate points with round scores for the 2016/2017 and 2017/2018 seasons in two separate sets of analyses. This approach was deemed to be reasonable because it allowed us to corroborate the results of our discovery data set (i.e., the season 2016/2017 jumps) with a replication data set (i.e., the season 2017/2018 jumps). Individual effect sizes were treated as independent in all our analyses (i.e., similar to a sample-wise *N* procedure; for a discussion, see [9]). This is reasonable because (i) all effect sizes were based on samples comprising identical participants (i.e., the competing athletes: with some allowance that needs to be made for disqualifications, non-participation of individual athletes in certain events, and athlete attrition in the second round of individual events), (ii) these participants represent the entire population of World Cup ski jumpers in the season 2016/2017 as well as 2017/2018, and consequently (iii) it can be largely ruled out that between-effect size heterogeneity of study effects was meaningfully affected by this approach.

Prior to all analytic procedures, primary effect sizes (i.e., the correlations between wind/gate points and total scores) were transformed to Fisher’s *Z*, following standard meta-analytic procedures [10]. Subsequent to the analyses, effect sizes were backtransformed to the *r* metric for ease of interpretation. In all our analyses, data points were weighted according to inverse study variances. First, we calculated meta-analytic summary effects by means of random-effects models. We interpret effect sizes according to the well-established classification of Cohen [11] into small, moderate, and large effects (lower thresholds correspond to absolute *r* = .10, .30, and .50, respectively). *I*-squared values are used as tentative indices for between-competitions heterogeneity using 25%, 50%, and 75% as the lower thresholds for small, moderate, and large heterogeneity, respectively [12]. Moreover, significance values of observed between-competitions heterogeneity were computed using chi-squared tests based on Cochran’s *Q*.

Second, we compared summary effects from (i) the first vs. second round as well as (ii) different competitive scoring systems (i.e., standard scoring of single jumps vs. team or KO competitions). Third, we used weighted mixed-effects meta-regressions to investigate potential influences of hill size on gate points and absolute wind points and round scores associations. Finally, we conducted sensitivity analyses by means of leave-one-out analyses to assess potential influences of single-study outliers (i.e., leverage points) on the observed summary effects. In this approach, *k* summary effects are calculated by omitting one primary effect in each turn which should not yield considerable changes between the resulting summary effects.

### Final sample

**Fig 1:**
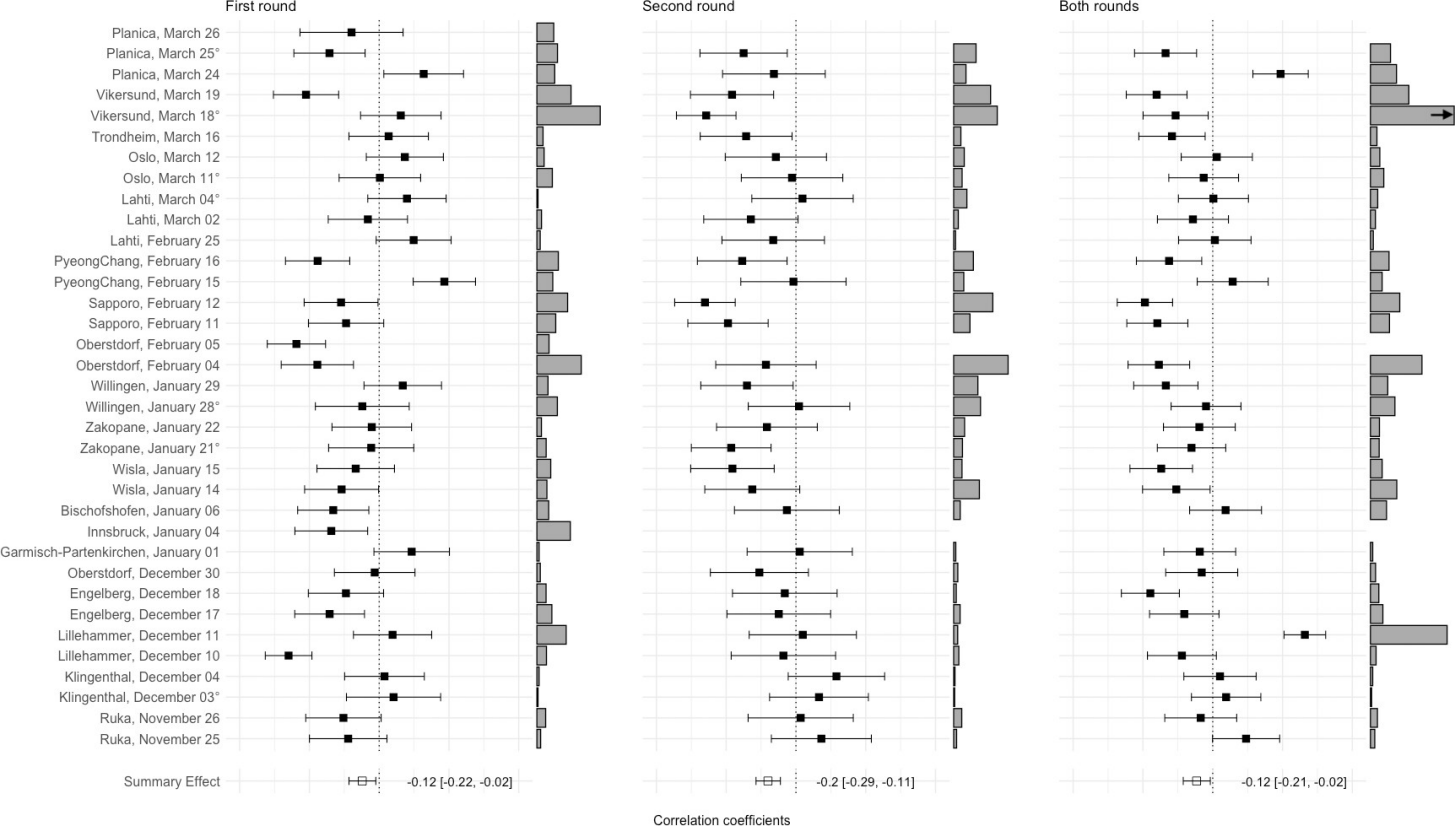
Forest plots of associations of wind points with round scores for first round, second round, and both rounds in the ski jumping season 2016/2017. Larger bars next to the effect sizes represent larger wind point variability and consequently less comparable wind conditions between competitors; the arrow indicates truncation of the wind point variability bar; for second round and both rounds results, no effect sizes are available for Garmisch Partenkirchen, January 01, Oberstdorf, February 05, and Planica, March 26 due to cancellation of the second round;° = team competition; “= KO competition; * = world championship.

For the 2016/2017 season, we included data from 35 ski jumping competitions (all 32 World Cup and three World Championship events), comprising 28 single (24 events with standard scoring; four KO competitions) and seven team competitions. In three competitions, the second round was cancelled due to adverse weather conditions which accordingly reduced the number of includable associations of scores with wind or gate points for second round and both rounds calculations to 32 (see, Note of Fig 1). The number of associations with gate points was further reduced, because in about a third of the respective rounds within competitions, all athletes started from the same gate.

For the 2017/2018 season, we included data from 30 World Cup competitions comprising 22 single (18 with standard scoring; four KO competitions) and eight team competitions. Includable results for second and both rounds calculations were reduced to 29 due to weather and gate point calculations were further reduced by a third because of constant starting gates. The number of included effect sizes and jumps are provided in the first two columns of Tables 1 and 2 (*k* range = 13 to 35; *N* range = 404 to 1950). All data are available from the electronic online supplement S1 Data.

**Table 1:** Associations of wind points with round scores.

	*k*	*n*	*I*^2^	τ^2^	*Q*	*r*	LCI	UCI	*ES*_min_	*ES*_max_	*p*
Season 2016/2017											
First round	35	1640	73.77	.064	129.16***	-.121	-.216	-.024	.005	.649	.014
Second round	32	975	47.51	.033	59.04***	-.200	-.286	-.112	.018	.652	< .001
Both rounds	32	1950	78.66	.064	143.47***	-.116	-.211	-.018	.005	.660	.021
Season 2017/2018											
First round	30	1391	72.77	.062	108.26***	-.119	-.219	-.014	.003	.673	.026
Second round	29	882	56.54	.048	64.43***	-.141	-.242	-.036	.024	.650	.009
Both rounds	29	1852	88.44	.126	272.91***	-.127	-.259	.009	.028	.765	.068

*I*^2^ = percentage of between effect variability because of true heterogeneity; τ^2^ = between effect variance due to true heterogeneity; *df*s for *Q*s = *k—*1, respectively; LCI = lower bound of 95% confidence interval; UCI = upper bound of 95% confidence interval; *ES*_min_ = smallest absolute individual effect size; *ES*_max_ = largest absolute individual effect size.

*** = *p* < .001.

**Table 2:** Mixed-effects subgroup analyses for wind and gate point effects according to scoring modes of different competition types.

	Wind points				Gate points			
	Standard scoring *r*	Other scoring *r*	*Q*	*p*	Standard scoring *r*	Other scoring *r*	*Q*	*p*
Season 2016/2017								
First round	-.143	-.067	0.661	.416	.307	.158	1.619	.203
Second round	-.189	-.212	0.050	.824	.379	.351	0.047	.828
Both rounds	-.129	-.088	0.256	.613	.350	.248	1.233	.267
Season 2017/2018								
First round	-.144	-.078	0.352	.553	.004	.244	1.006	.316
Second round	-.153	-.124	0.077	.782	.203	.181	0.029	.866
Both rounds	-.117	-.143	0.027	.870	.176	.365	2.607	.106

Standard scoring = Single competition; Other scoring = KO and team competitions; *Q* = weighted sum of squared differences between individual study effects and pooled study effect (Cochrans’*Q*); *df* = 1 for all analyses.

## Results

### Wind points

For the 2016/2017 season, associations of round scores with wind points showed negative correlations which were most pronounced for second round jumps, yielding a small-to-moderate effect (*r* = -.196; *p* < .001). Interestingly, *I*-squared values were high, indicating (significant) moderate-to-large heterogeneity between competitions. This is an important observation, because it indicates that in individual competitions, wind points can affect total scores to a stronger extent, than the summary effect suggests. For instance, on average a moderate 4% of the variance of round scores were explained by wind points in round two (i.e., corresponding to the observed summary effect), but the variance explanation of wind points in single competitions reached up to 42% (i.e., corresponding to the largest observed effect size in this round; see penultimate column in the top entries of Table 1).

Results for the 2017/2018 season were virtually identical in terms of effect direction and strength, although associations for both rounds failed to reach nominal statistical significance. However, effect sizes showed robust negative associations between wind points and round scores as well as substantial between-competition heterogeneity, indicating up to 58% of variance explanations in round scores due to wind points (see bottom entries of Table 1). All round-specific results are provided in the upper half of Table 1 and forest plots for individual competitions are available from Fig 1.

A subgroup analysis between first and second round associations did not show nominally significant differences (*Q*(1) = 1.251; *p* = .263) between the two for the 2016/2017 season. Further subgroup analyses showed no influences of competition scoring modes on associations between wind points and round scores. Effect sizes of competitions with standard scoring mode (i.e., single, non-KO competitions) were not significantly different from competitions with team or KO scoring (see, top left portion of Table 2). Examination of potential influences of the hill size on absolute wind points and round scores associations yielded consistently positive effects on coefficients indicating 22.53% (first round; *b* = 0.002; *p* = .043), 25.69% (second round; *b* = 0.002; *p* = .119), and 24.09% (both rounds; *b* = 0.002; *p* = .022) of explained variance due to hill size.

**Fig 2:**
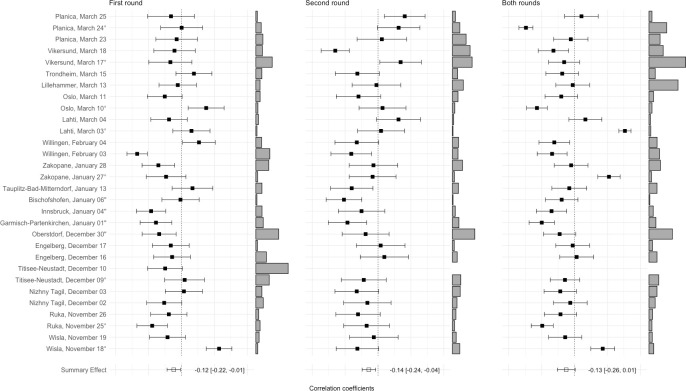
Forest plots of associations of wind points with round scores for first round, second round, and both rounds in the ski jumping season 2017/2018. Larger bars next to the effect sizes represent larger wind point variability and consequently less comparable wind conditions between competitors; for second round and both rounds results, no effect sizes are available for Titisee-Neustadt, December 10 due to cancellation of the second round;° = team competition; “= KO competition.

Round-specific results for the 2017/2018 season are provided in the bottom half of Table 1 and individual competition forest plots are available from Fig 2. Subgroup analyses were consistent with the 2016/2017 results, indicating no effects of round (*Q*(1) = 0.134; *p* = .715) or competition scoring mode (see bottom left portion of Table 2). However, findings for influences of hill size on coefficients yielded not entirely equivocal results, showing the expected positive significant association for second round (*b* = 0.002; *p* = .003) but negative (albeit non-significant) associations for first (*b* = -0.002; *p* = .073) and both rounds results (*b* = -0.001; *p* = .541).

In supplementary analyses, we examined the influence of wind compensation point variability on associations between wind points and round scores by predicting absolute primary effect sizes through standard deviations of within-competition wind compensation points in weighted mixed-effects meta-regressions. The signs of the regression coefficients were consistent across first, second, and both rounds scores (excepting the both rounds analysis for the 2017/2018 season), indicating a positive relationship between wind points variability and the strength of the effect sizes (Table 3). Finally, sensitivity analyses did not show substantial influences of single studies on our summary effect estimates in either season.

**Table 3:** Mixed-effects meta-regressions of absolute effect sizes for wind point associations on standard deviations of wind points within competitions.

	*k*	*R*^2^	*I*^2^	*b*	*SE*	*p*
	Season 2016/2017					
First round	35	21.40	27.37	0.037	0.020	.076
Second round	32	100.00	0.00	0.064	0.021	.002
Both rounds	32	34.87	40.12	0.031	0.011	.005
	Season 2017/2018					
First round	30	<0.01	37.18	0.020	0.023	.386
Second round	29	-	<0.01	0.023	0.025	.369
Both rounds	29	0.06	76.64	-0.028	0.029	.331

*I*^2^ = percentage of remaining between effect variability because of true heterogeneity; *R*^2^ = percentage of between effect heterogeneity that is accounted for by wind points variability; empty cell entries in the *R*^2^ column indicate unavailability of estimates due to residual tau-squared values ~ 0.

### Gate points

Associations of round scores with gate points were consistently positive and most pronounced for second round results, yielding a moderate effect (*r* = .370; *p* < .001; ~14% of explained variance) in our 2016/2017 season data. Heterogeneity between competitions was significant in all calculations, yielding moderate to high values. This points once more to a large variability of effect sizes, indicating up to about 64% of variance explanation within single competitions (see, penultimate column of the top half of Table 4).

**Table 4:** Associations of gate points with round scores.

	*k*	*n*	*I*^2^	τ^2^	*Q*	*r*	LCI	UCI	*ES*_min_	*ES*_max_	*p*
Season 2016/2017											
First round	23	1045	81.07	.101	115.11***	.263	.124	.392	.049	.674	< .001
Second round	18	552	59.23	.053	41.68***	.370	.245	.482	< .001	.803	< .001
Both rounds	23	1408	73.37	.047	82.43***	.310	.214	.401	.017	.777	< .001
Season 2017/2018											
First round	18	811	77.11	.080	75.82***	.195	.048	.334	.023	.614	.010
Second round	13	404	76.66	.117	50.14***	.155	-.056	.353	.089	.641	.149
Both rounds	18	1194	81.67	.070	89.87***	.275	.145	.395	.015	.596	< .001

*I*^2^ = percentage of between effect variability because of true heterogeneity; τ^2^ = between effect variance due to true heterogeneity; *df*s for *Q*s = *k—*1, respectively; LCI = lower bound of 95% confidence interval; UCI = upper bound of 95% confidence interval; *ES*_min_ = smallest absolute individual effect size; *ES*_max_ = largest absolute individual effect size.

* = *p* < .05

** = *p* < .01

*** = *p* < .001.

Summary effects did not differ significantly between first and second round results (*Q*(1) = 1.423; *p* = .233) and in regard to competition scoring modes (see top right portion of Table 2). Hill sizes were consistently positively related to observed effect sizes, suggesting larger gate points and round scores associations for competitions on larger hills, although regression coefficients only reached nominal significance for both rounds (*b* = 0.003; *p* = .016; 24.34% explained variance), but not first (*b* = 0.001; *p* = .651; < 0.01% explained variance), or second round (*b* = 0.002; *p* = .343; < 0.01% explained variance).

Round score and gate points associations of our season 2017/2018 analyses were again consistently positive, although they were less pronounced (see bottom half of Table 4) and between-competitions heterogeneity was large. First and second round summary effects did not differ significantly (*Q*(1) = 0.489, *p* = .484). However, results for influences of hill sizes on coefficients contrasted the 2016/2017 results, yielding negative associations in all analyses (both rounds: *b* = -0.003, *p* = .119, 16.41% explained variance; first round: *b* = -0.002, *p* = .177, 9.63% explained variance; second round: *b* = -0.005, *p* = .005, 49.16% explained variance). Sensitivity analyses did not indicate meaningful influences of leverage points on summary effects in either season.

## Discussion

In all, we show that round points are negatively associated with wind points but positively associated with gate points. In general, the heterogeneity of effect sizes between individual competitions was high for both compensation mechanisms. These findings present several points of interest, as discussed below.

Wind compensation points were negatively related to round scores across all analyses (i.e., first round vs. second round vs. both rounds), which indicates systematic undercompensation in unfavorable wind conditions. Summary effects were observed to be moderate, accounting for up to 4% of variance in the observed wind and round scores associations for all competitions. These results are in line with past findings that indicated that awarded wind compensation points are typically too low [7, 8]. The observed effects were stronger for second round than for first round results, indicating stronger undercompensation for athletes that obtain higher classifications, although this difference did not reach nominal statistical significance. These findings may be interpreted as functions of athlete ability, because athletes that qualify for the second round may be expected to yield more homogeneous jumps, which exaggerates the effects of undercompensation. This pattern of results remained stable when results were subgrouped according to different scoring modes of competitions, thus demonstrating robustness of the observed effects.

Perhaps of even more importance is our observation of the substantial heterogeneity (including sign changes) of effect sizes between different competitions. Our observation of about 48% to 88% true heterogeneity (i.e., heterogeneity that is due to systematic variation, not sampling error) between competitions indicates that the wind compensation points do not affect round scores in an identical fashion in different competitions. This means that although wind points moderately undercompensate round scores on average, these effects are not equally distributed over all competitions of a season.

For instance, as can be seen in Fig 1, the smallest observed primary effect for second round results showed a trivial correlation, yielding *r* = -.018 (PyeongChang, February 15), consequently leaving jumpers results virtually unaffected by wind malcompensation. In contrast, the largest observed primary effect in this subgroup was *r* = -.652 (Sapporo, February 12) which is equivalent to about 43% of explained variance of the round scores due to wind compensation points. This means that more than a third of the variation between the jumpers is not due to the differing jumper’s ability, but rather due to the differing wind conditions and therefore awarded compensation points. Consequently, the assumption that effects of wind conditions will even themselves out over the ski jumping season may not be entirely justified. Perhaps even more critically, this means that the results of isolated important events such as the World Championships may be considerably affected by unfavorable conditions.

Obviously, a portion of these differences in the strength of the relationships between wind points and round scores may be due to the variance in awarded wind points. This means that when wind conditions are comparable for all or most of the athletes (i.e., in cases where awarded wind points only vary to a minor degree), compensation points are bound to show comparatively small influences on round scores. In fact, our examination of influences of wind point variability on observed effect sizes showed substantial influences of wind point variability on the round score and wind points association. These results indicate that wind points appear to yield fair compensations in competitions where wind conditions are comparable across jumps, whilst unstable weather conditions lead to increasingly unreliable (i.e., too low) point compensations.

On the one hand, these findings may be seen as support for the idea that wind points “probably ‘correct[s]’ most cases where wind plays an import role [in steady wind conditions]” ([7] p.368). On the other hand, wind points were designed to serve as a mechanism that should render athletes’ performance comparable in unsteady wind conditions. In the light of our results, it seems questionable whether this expectation can be satisfactorily met by the current system, because higher wind point variability leads to less accurate compensations. In other words: Wind points provide fair compensations only when they are not needed.

Moreover, there was some evidence for influences of hill size on the reliability of wind compensation points. Specifically, hill size predicted wind points and round scores associations positively, suggesting larger inaccuracies of the currently used compensation point system for larger hills. Of note, the variable hill size is included in the wind compensation formula, but does not lead in its current form to appropriate compensations. Importantly, according to our sensitivity analyses, the observed large between-competitions variability is not due to untypical results that may be attributable to leverage points. Our results remained stable even when individual effects were excluded from summary effect estimations.

Gate points were positively related with round scores. These results were to be expected, because coaches are allowed to deliberately reduce the inrun length to prevent overly long and therefore dangerous jumps of strong athletes. This means that the observed moderate positive associations may to a certain extent be seen as a result of stronger athletes starting from lower gates. Once more, the observed relationships were strongest for second round results, suggesting larger associations for the best-performing jumpers and accounting for about 14% of variance. This may indicate that a certain portion of the positive gate points and round scores association is not due to athlete performance-related coaches’ decisions, but rather represent overcompensations of gate points (i.e., if athlete performance and coaches decisions were the only contributing factor, first round results should have yielded larger effect sizes because athlete performance variability is higher). This interpretation is in line with previous findings that suggested about 10% overcompensation of gate points [8].

The variability of effect sizes between competitions was large, indicating 59% to 82% true heterogeneity. This shows that round scores are not affected in an identical fashion by gate points across different competitions. However, it should be acknowledged that the variability in gate points is comparatively low by default, because (i) gate changes are compensated with a fixed number of gate points (i.e., reduction of the inrun length by one gate always yields the same number of gate points within a given competition, although the values may vary between competitions according to hill size) and (ii) gate points are often awarded to a comparatively small number of athletes because a majority may opt to start from an identical starting gate. These factors may contribute to the large variability between effect sizes.

An examination of different scoring modes did not show significant differences between effect sizes according to standard and team or KO scoring, thus indicating robustness of the observed associations. Influences of hill size on associations between gate points and round scores could not be reliably determined because coefficient signs differed considerably between the two examined seasons, thus leaving potential effects to be clarified. Finally, sensitivity analyses did not show meaningful influences of single competitions on summary effects, thus corroborating effect stability of all our bivariate associations.

### Limitations

Three limitations of this study need to be acknowledged. First, we did not investigate potential non-linearity of wind point effects on round scores. It has been shown, that effects of wind on jump length are not linear [7], which may have led to an underestimation of summary effects in the present study. However, we showed a robust meaningful negative association of wind points with round scores which may be interpreted as a lower threshold of the underestimation of wind compensation points. It needs to be acknowledged that between-competition heterogeneity was large, so that isolated results of some competitions would even indicate overcompensating effects of wind points. Notwithstanding, although potential effects of peculiarities of individual hills cannot be ruled out, negative wind points and round scores associations prevailed throughout competitions and seasons. Second, effects of sideways wind gusts on round scores could not be investigated, because data is currently unavailable (i.e., sideways gusts are not measured on jumping hills). Finally, based on the present design it cannot be clearly decided whether performance-related coaches decisions or overcompensation are responsible for gate point correlations with round scores. However, based on the presently observed comparatively large effects and in agreement with previous observations of gate point overcompensations [8], it seems likely that performance-related inrun length reduction is not the sole cause for the observed positive correlations.

### Conclusions

Taken together, our examination of wind points and round scores associations indicates that wind compensation points are systematic underestimates of the necessary corrections for changing wind conditions in ski jumping. These findings may be of particular relevance for isolated events, such as the World Championship or the Olympics. Especially, unsteady wind conditions appear to exacerbate problems of the established compensation systems. Apparently, these compensation systems become increasingly unreliable when they are most necessary. However, the large variability of wind points between events–which in turn affects wind points and scores correlations–shows that suboptimal compensation mechanisms may conceivably exert meaningful effects even on seasonal World Cup results. Moreover, we showed tentative evidence for overcompensation of gate points.

These findings have implications for policy makers, event organizers, and athletes alike. First and foremost, the formula that is currently used to calculate wind compensation points should be reevaluated. The concept of wind compensation points aims at correcting unfair results due to differing conditions, which renders the present performance of this system (i.e., increasingly unreliable compensations when weather conditions become unsteady; systematic undercompensation) unsatisfactory. Potential moderating properties of hill size beyond its current representation should be accounted for in a revision of the adopted formula. The positive association between hill size and associations of wind points with round scores suggests that the constant (i.e., the value “36”) that is currently deducted from the variable hill size in the wind points formula is too small. Obviously, an alternative to such a reevaluation would be the introduction of more conservative standards in terms of weather conditions that are deemed suitable for the start and continuation of competitions. However, such a measure would come at the cost of an increasing number of cancelled or delayed competitions.

Second, considering the substantial influence of wind on athlete performance it seems sensible to aim at a more reliable assessment of wind speeds at different sections of the jumping hills. The current approach is to measure winds on either 5, 7 or 10 points of the slope on normal, large, and ski-flying hills, respectively which corresponds roughly to a spacing of 20 meters in-between measurement points.

On the one hand, increases of the number of measurement points may increase the reliability of measured wind speeds and consequently the accuracy of wind points. A gold standard has been suggested by Müller [13] who argued that anemometers set at every 5 to 10 meters of the glide path may assist in providing appropriate compensations through computer simulations for individual competitions.

On the other hand, the introduction of a more reasonable weighting procedure for the tangential wind speed according to the measurement point may further improve wind point accuracy. This may be sensible because (un-)favorable winds affect the length of a jump in a different way at the beginning than at the end of a jump. Jung and colleagues [2] showed that head winds give an advantage only in the later phase of a jump but are disadvantageous in the initial phase (for tail winds, it is the other way around). Our observation of the non-trivial negative association between wind points and scores suggests that advantages of head winds in the later phase of the jump outweigh their disadvantages at the start and disadvantages of tail winds in the later phase of the jump outweigh their advantages at the start, when assuming that the wind comes, more often than not, from the same direction during a single jump (i.e., most jumpers will have either head or tail wind only within one jump).

Although wind speeds are weighted according to the different measurement points, assigning higher weights to wind speeds in the middle and the second part of the flight, (dis-)advantages because of (head) tail winds in the early phase of the flight are not accounted for. It seems necessary to revise the current weighting system to account for these differential influences.

Ideally, it would be possible to include head, tail, and crosswinds in the compensation system to calculate wind influences in a 3D computer model that allows for more accurate compensations in real time [2]. However, pursuing this option would come at the cost of reducing attractiveness for the spectators, because of the increased complexity of determining the jumpers’ performance.

In a similar vein, establishing more protective equipment against sideways wind gusts (such as nets or walls, which have been installed on a number of, but not all, hills) as already suggested by Müller [13] may be sensible to facilitate comparability of results.

Finally, as long as the current system is in effect, athletes of comparable performance levels (or their coaches) may wish to opt sooner rather than later for lower starting gates, if other athletes’ jumps approach distances of the K-point. Even when some allowance is being made for moderating influences of athletes’ performance, it seems likely that lower starting gates (and consequently higher gate compensation points) are related to better round scores.

## Supporting information

S1 Checklist PRISMA checklist. (DOC)

S1 Fig PRISMA flow-chart. (DOC)

S1 Data Data for first, second and both round correlations. (SAV)
