# Supplementary material for: After all, it is an outdoor sport: Meta-analytic evidence for negative associations between wind compensation points and round scores in ski jumping competitions
Source: PLoS One. 2020 Aug 24;15(8):e0238101. doi: 10.1371/journal.pone.0238101 (PMC7446911; doi:10.1371/journal.pone.0238101)
Supplement: S1 Fig — (DOC) [file pone.0238101.s002.doc]

**Screening**

**Included**

**Eligibility**

**Identification**

Records identified through the FIS database
(*k* = 35 for 2016/2017 and

*k* = 30 for 2017/2018 season))

Records after duplicates removed
(*k* = 65)

Records screened
(*k* = 65)

Records excluded
(*k* = 0)

Extracted full records
(*k* = 65)

Records included in quantitative synthesis

(*k* = 65)

Records excluded
(*k* = 0)
